# Change in walking cadence as a digital outcome measure of clinically meaningful improvement in gait speed and 6-minute walk test distance after a mobility intervention in older adults

**DOI:** 10.1371/journal.pone.0337414

**Published:** 2026-05-29

**Authors:** Daniel S. Rubin, Nancy W. Glynn, Marcin Straczkiewicz, Margaret K. Danilovich, Christopher Sciamanna, Jennifer S. Brach

**Affiliations:** 1 Department of Anesthesia and Critical Care, University of Chicago, Chicago, Illinois, United States of America; 2 Department of Epidemiology, School of Public Health, University of Pittsburgh, Pittsburgh, Pennsylvania, United States of America; 3 Department of Measurement and Electronics, AGH University of Krakow, Krakow, Poland; 4 Department of Physical Therapy and Human Movement Sciences, Northwestern University, Chicago, Illinois, United States of America; 5 Department of Medicine, Penn State University, Hershey, Pennsylvania, United States of America; 6 School of Health and Rehabilitation Sciences, University of Pittsburgh, Pittsburgh, Pennsylvania, United States of America; University of Illinois Urbana-Champaign, UNITED STATES OF AMERICA

## Abstract

Mobility assessments are essential for evaluating baseline function and monitoring responses to interventions in older adults. Usual-pace gait speed and the 6-minute walk test (6MWT) are widely used and reproducible measures with established minimum clinically important differences (MCIDs) to distinguish responders from non-responders. However, both require in-person administration, limiting their scalability in clinical trials and population-based studies. Walking cadence, a measure of walking intensity that can be captured digitally, may offer a scalable alternative for identifying responders versus non-responders to mobility interventions. We conducted a secondary analysis of the prospective Program to Improve Mobility in Aging (PRIMA) cohort trial to evaluate whether changes in walking cadence after a walking intervention could identify responders versus non-responders. Cadence was measured during usual-pace gait speed testing and the 6MWT, and logistic regression models assessed its ability to predict achievement of MCIDs for gait speed (>0.1 m/s) and 6MWT distance (>30 m) in older adults. Data from 213 participants were analyzed. Change in median walking cadence predicted improvement in usual-pace gait speed with an area under the curve (AUC) of 0.90 (95% CI: 0.85–0.94). The Youden Index identified an increase of ≥3 steps/min as the optimal threshold (sensitivity 0.81; specificity 0.88). For predicting improvement in 6MWT distance, the AUC was 0.80 (95% CI: 0.74–0.86), with the same ≥3 steps/min threshold (sensitivity 0.75; specificity 0.77). These findings suggest that changes in walking cadence during usual-pace gait and the 6MWT may serve as a digitally measurable outcome to identify responders to mobility interventions. Further research is warranted to validate these findings in remote and real-world applications.

## Introduction

Mobility assessments are essential tools for evaluating baseline function and monitoring response to interventions. Two commonly used performance-based tests are the usual pace gait speed and the 6-minute walk test (6MWT)[1,2]. Usual pace gait speed reflects overall mobility and is a strong predictor of health outcomes, while the 6MWT measures walking endurance and functional capacity [2,3]. Both tests are widely used in clinical trials and large-scale epidemiologic studies to track changes in function over time. However, a major limitation of these assessments is their reliance on in-person administration [4,5]. Travel to clinics or research sites can be particularly burdensome for frail older adults, leading to lower participation rates and reduced frequency of assessment. Additionally, these tests require trained personnel, further increasing costs and logistical barriers. Despite the clinical utility of usual pace gait speed and the 6MWT, scalable and remote methods for assessing functional improvement are lacking.

One potential solution is to measure alternative walking parameters, such as cadence—the number of steps per minute—as a remote-friendly metric rather than speed or distance [6,7]. Cadence is a fundamental parameter of walking that reflects walking intensity and can be reliably measured using wearable devices [6–9]. Unlike GPS-based measurement of distance or speed—which is often inaccurate indoors and impractical for older adults lacking access to safe outdoor spaces—changes in walking cadence can be accurately measured indoors using wrist- or hip-worn accelerometers. Wearable devices, including smartwatches or smartphones, can deliver standardized walking tests remotely (e.g., 6MWT) and measure changes in cadence as an objective response to an intervention. Prior work has examined cadence as a target to guide walking intensity or as an independent outcome of an intervention; however, evaluating changes in cadence during standardized mobility assessments may provide a more direct assessment of functional improvements when traditional in-person assessments are impractical.

Despite its promise, it remains unknown whether changes in cadence measured during usual-pace walking or during a 6MWT can reliably identify individuals who achieve clinically meaningful improvements in function. The minimum clinically important difference (MCID) provides a validated framework for defining such change, with validated thresholds including an increase of 0.1 m/s for gait speed and 30 meters for the 6MWT [10]. These thresholds are used in clinical trials and rehabilitation programs to define treatment response and guide clinical decision-making. Applying an MCID-based, responder/non-responder framework to changes in cadence would represent an important advance, as it moves beyond descriptive measurement toward a clinically interpretable digital endpoint that can be used to evaluate intervention efficacy in remote and scalable settings.

The Program to Improve Mobility in Aging (PRIMA) study was a prospective randomized trial of a structured exercise intervention in older adults with mobility limitations [11,12]. The intervention combined the Group Lifestyle Balance program with individualized physical therapy and assessed usual pace gait speed, 6MWT distance, and walking cadence at multiple time points. In this secondary analysis of the PRIMA cohort we evaluated changes in walking cadence among older adults following the 12-week exercise intervention to assess whether changes in cadence can reliably identify participants who improved above the MCID for usual pace gait speed and 6MWT. By evaluating the utility of cadence as a marker of functional improvement, this study may support future efforts to remotely measure walking performance in older adults—offering a scalable and accessible approach for both clinical practice and research.

## Methods

### Study design

We conducted a retrospective secondary data analysis to investigate the association between changes in walking cadence and improvements in usual pace gait speed and 6MWT distance following a mobility intervention (PRIMA: NCT02663778) [11]. Briefly, participants were aged at least 65 years of age, ambulatory without assistance, had a usual gait speed of > 0.60 to < 1.2 m/s as measured as the average from two trials of a 4-meter gait speed, and physician clearance to exercise [11]. The primary study was conducted between April 1^st^ 2016 and January 1^st^ 2020. Participants were randomized to a two-arm trial comparing a standard strength, endurance, and flexibility program with a combined program that added timing and coordination training, to evaluate effects on mobility activity and participation in community-dwelling older adults. Both groups also received a behavioral physical activity intervention based on the Group Lifestyle Balance program [11]. Complete details and primary outcomes of the intervention have been previously published [11,12]. Importantly, no significant differences were found between the two intervention arms. The University of Pittsburgh and the University of Chicago institutional review boards both approved the study. The approval number is STUDY19040153. All participants provided written informed consent.

### Study sample and data collection

Participants were assessed at the time of enrollment (baseline) and at 12-, 24- and 36-weeks following randomization. For this secondary data analysis, we only used the baseline and 12-week data as the primary aim of our analysis was to identify responders to the intervention as measured by usual pace gait speed and the 6MWT. Study procedures included demographics and medical history via questionnaire. Objective measures of physical function included usual pace gait speed, Short Physical Performance Battery (SPPB), and 6MWT [13–15]. Assessments were conducted by a trained physical therapist, masked to group assignment, following standardized testing procedures for all measures.

### Assessments

#### Usual pace gait speed cadence.

Cadence was measured using a validated method during usual, self-selected walking speed over an instrumented walkway (Protokinetics LLC, Havertown, PA) [11]. After instruction and two short practice walks, participants completed six passes at their usual pace. Walking cadence (steps/min) and gait speed (m/s) were averaged over the six passes.

#### 6MWT cadence.

During the 6MWT, participants wore an ActiGraph GT3X+ accelerometer (ActiGraph, Pensacola, FL) on the non-dominant wrist, set to record raw acceleration data at 30 Hz. Data from the accelerometer was downloaded and the 6-minute walking segment was identified and extracted to be used for the cadence analysis. Adaptive Empirical Pattern Transformation method (ADEPT) was used to analyze the raw accelerometer output from the accelerometer [16]. The ADEPT algorithm has been previously validated and is freely available for download as ADEPT R package [17]. We estimated the walking cadence (steps/min) for each of the six minutes of the 6MWT. Finally, an overall median of cadence (steps/min) was computed for each 6MWT [16].

### Outcome

The primary outcome was functional improvement above the MCID. For usual pace gait speed, an MCID of ≥0.1 m/s was used, and for the 6MWT, an MCID of ≥30 meters [10,18]. Participants were categorized as “responders” if they improved above the respective MCID at the 12-week assessment, which marked the completion of the intervention.

Given the variability in MCID thresholds for gait speed and 6MWT in older adults, we also conducted sensitivity analyses. These included a less conservative threshold of ≥0.05 m/s for gait speed, and both a more stringent (≥50 meters) and less stringent (≥20 meters) threshold for 6MWT distance [10]. We selected the primary MCID thresholds of ≥0.1 m/s and ≥30 meters based on prior studies in older adults with mobility limitations and comorbidities, such as those represented in the PRIMA cohort [10,19].

### Statistical analysis

Descriptive statistics (means, standard deviations, medians, interquartile ranges, and frequencies) were calculated for demographic, clinical, and functional variables. Between-group comparisons were made using Mann-Whitney U test and Chi-square tests where appropriate.

Pearson correlation coefficients were used to assess the association between changes in walking cadence and changes in gait speed and 6MWT distance. Univariate linear regression models were used to evaluate the association between changes in cadence and changes in the two outcome measures. The independent variable was change in cadence; dependent variables were change in gait speed and 6MWT distance.

To evaluate the predictive utility of cadence changes for identifying responders, we used univariate logistic regression models. The dependent variables were binary indicators of improvement above the MCID for gait speed and 6MWT distance and the primary predictor was change in cadence. We selected a univariable modeling approach to directly quantify the relationship between cadence and clinically meaningful changes in walking performance, consistent with the primary objective of establishing cadence as a proxy measure of functional improvement. Given the modest sample size and the focus on evaluating cadence as a standalone digital endpoint, this approach minimizes overfitting and preserves interpretability without adjustment for additional covariates. Model performance was assessed using receiver operating characteristic (ROC) curves, and optimal cut points were identified using the Youden Index to maximize sensitivity and specificity.

We performed two different sensitivity analyses. First, we evaluated whether the association between changes in cadence and functional improvement were robust to alternative MCID thresholds for both gait speed (0.05 m/s) and 6MWT distance (20 meters and 50 meters). Second, we assessed whether model performance improved with a multivariable approach by incorporating covariates likely to influence walking performance, including age, body mass index, sex, and baseline values for usual-pace gait speed and 6MWT distance, respectively. All statistical tests were two-sided with an alpha level of 0.05. Accelerometer data were analyzed using R software, and all other statistical analyses were conducted using STATA version 16 (StataCorp, College Station, TX).

## Results

A total of 249 participants were enrolled in PRIMA, and 213 had complete data for both usual pace gait speed and 6MWT at baseline and 12 weeks. Participant characteristics are shown in Table 1. The mean age was 77 ± 6 years, 65% (139/213) were female, the baseline usual pace gait speed was 1.0 ± 0.2 m/s and the mean 6MWT distance was 407 ± 84 meters.

**Table 1:** Patient Characteristics.

Variables	N = 213 Mean ± Standard Deviation or n (%)
Age, years	77.3 ± 6.4
Sex	
Male	74 (34.7)
Height, inches	65.5 ± 4.0
Weight, pounds	173.9 ± 37.6
Body Mass Index, kg/m^2^	28.6 ± 5.8
Race	
Asian	1 (0.5)
Black	13 (6.1)
Native Hawaiian or Other Pacific Islander	2 (0.9)
White	192 (90.1)
Other	1 (0.5)
Refused to say	4 (1.9)
Cardiac Disease	18 (8.5)
Neurological Disease	13 (6.1)
Musculoskeletal Disease	184 (86.4)
Diabetes	42 (19.7)
Cancer	78 (36.6)
Lung Disease	53 (24.9)
Duke comorbidity index	2.8 ± 1.2
Gait Speed, m/s	1.0 ± 0.2
Short Physical Performance Battery, 0–12	9.9 ± 1.6
Five sit to stand, seconds	13.5 ± 3.9
Six Minute Distance, meters	406.9 ± 84.4

### Changes in walking cadence during usual pace gait speed

At baseline, participants who improved their usual pace gait speed by ≥0.1 m/s (“responders”) had a slightly lower median walking cadence compared to non-responders (108 [IQR: 103–113] steps/min vs. 111 [IQR: 106–116] steps/min; *p* = 0.01) (Table 2). Over 12 weeks, responders showed a greater median increase in walking cadence than non-responders (8 [IQR: 5–11] steps/min vs. 0 [IQR: −3–3] steps/min; *p* < 0.001). Change in walking cadence was strongly correlated with change in usual pace gait speed (r = 0.87, 95% CI: 0.84–0.90; *p* < 0.001). Each 1 step/min increase in walking cadence during usual pace gait speed testing was associated with a 0.05 m/s (95% CI: 0.04–0.06) increase in gait speed.

**Table 2:** Cadence during usual pace gait speed.

Variables: Median (IQR)	Non-responders (n = 128)	Responders (n = 85)	Total (n = 213)	P-value
Baseline usual pace gait speed (meters/second)	1.1 (1.0 - 1.2)	1.1 (0.9 - 1.2)	1.1 (1.0 - 1.2)	0.060
Post-intervention usual pace gait speed (meters/second)	1.1 (1.0 - 1.2)	1.2 (1.1 - 1.4)	1.2 (1.0 - 1.3)	<0.001
Mean change in usual pace gait speed	0.0 (−0.0 - 0.1)	0.2 (0.1 - 0.3)	0.1 (0.0 - 0.2)	<0.001
Baseline median cadence (steps/min)	111 (106 - 116)	108 (103 - 113)	110 (105 - 115)	0.01
Post-intervention median cadence (steps/min)	112 (106 - 117)	115 (111 - 122)	113 (108 - 119)	<0.001

**Fig 1:**
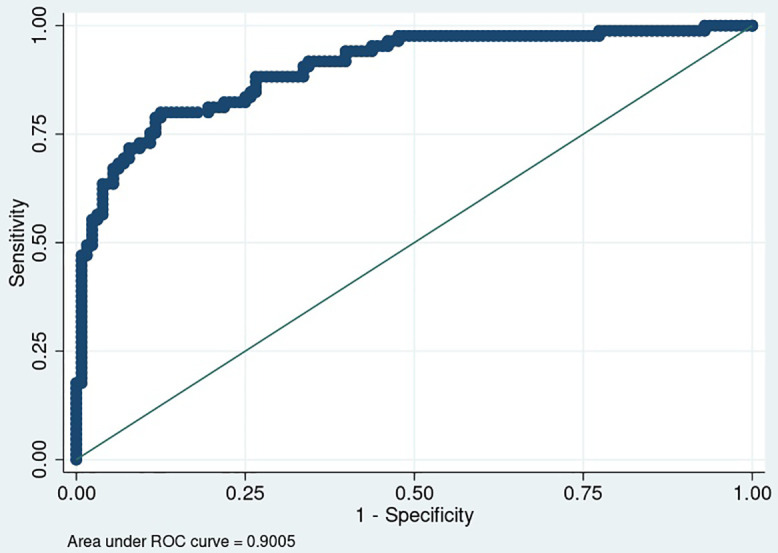
Area under the receiver operating characteristic curve for change in median walking cadence to predict improvement in usual pace gait speed ≥0.1 m/s.

Logistic regression analysis (Table 3) using change in median walking cadence to predict improvement in usual pace gait speed ≥0.1 m/s yielded an area under the AUC of 0.90 (95% CI: 0.85–0.94) Fig 1. The Youden Index identified an increase of ≥3 steps/min as the optimal threshold, with a sensitivity of 0.81 and specificity of 0.88 for detecting gait speed responders.

**Table 3:** Univariate Logistic Regression.

Dependent variable: Usual Pace Walk Test (MCID >0.1 m/s)			
Predictor	Odds Ratio (per 1 step/min increase)	95% (Confidence Interval)	P-value
Change in cadence during usual-pace walk test	1.54	1.36, 1.73	<0.01
Intercept	0.12	0.06, 0.22	<0.01
Model AUC	0.90	0.85, 0.94	–

Dependent variable: 6-minute walk test (MCID > 30meters)			
Predictor	Odds Ratio (per 1 step/min increase)	95% (Confidence Interval)	P-value
Change in cadence during 6-minute walk test	1.17	1.10, 1.24	<0.01
Intercept	0.68	0.48, 0.98	0.05
Model AUC	0.80	0.74, 0.86	–

### Changes in walking cadence during 6MWT

At baseline, there was no statistically significant difference in walking cadence between those who improved their 6MWT distance by ≥30 meters (“responders”) and non-responders (111 [IQR: 105–116] steps/min vs. 113 [IQR: 106–119] steps/min; *p* = 0.09) (Table 4). Over 12 weeks, responders demonstrated a greater median increase in walking cadence compared to non-responders (6 [IQR: 3–10] steps/min vs. 1 [IQR: −2–3] steps/min; *p* < 0.001). Change in walking cadence was modestly correlated with change in 6MWT distance (r = 0.37, 95% CI: 0.25–0.48; *p* < 0.001). Each 1 step/min increase in walking cadence during the 6MWT was associated with a 3.7-meter (95% CI: 2.7–4.7) increase in 6MWT distance.

**Table 4:** Cadence during 6-minute walk test.

Variables Median (IQR)	Non-responders (n = 99)	Responders (n = 114)	Total (n = 213)	P-value
Baseline 6-minute walk distance (meters)	405 (354 - 460)	401 (346 - 455)	405 (350 - 458)	0.199
Post-intervention 6-minute walk distance (meters)	413 (358 - 466)	478 (435 - 532)	445 (383 - 514)	<0.001
Median change in 6-minute walk distance	5 (−19 - 21)	62 (46 - 90)	33 (7 - 69)	<0.001
Baseline median cadence (steps/min)	113 (106 - 119)	111 (105 - 116)	111 (106 - 117)	0.09
Post-intervention median cadence (steps/min)	113 (107 - 120)	117 (111 - 122)	116 (109 - 122)	0.02

**Fig 2:**
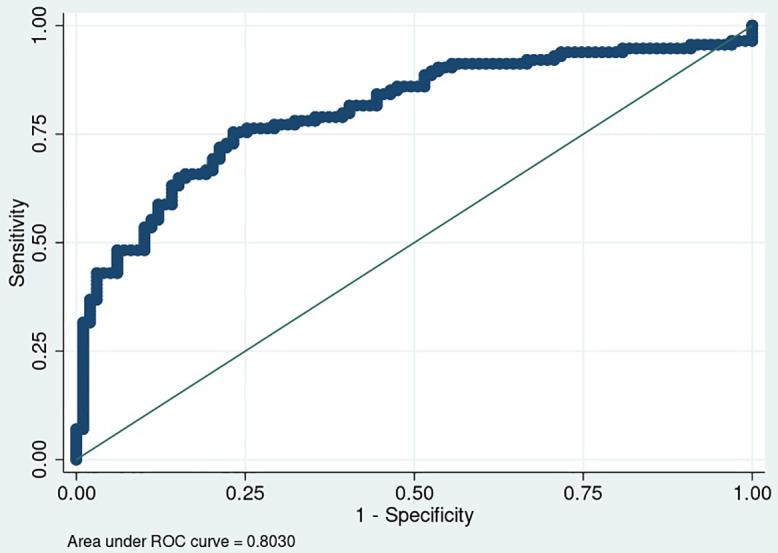
Area under the receiver operating characteristic curve for change in median walking cadence to predict improvement in 6-minute walk test distance by ≥30 meters.

Logistic regression analysis (Table 3) using change in median walking cadence to predict improvement in 6MWT distance ≥30 meters yielded an AUC of 0.80 (95% CI: 0.74–0.86) Fig 2. The optimal cut point identified by the Youden Index was an increase of ≥3 steps/min, with a sensitivity of 0.75 and specificity of 0.77.

### Sensitivity analysis

Sensitivity analyses using alternative thresholds for clinically meaningful change yielded results consistent with the primary analyses. For usual pace gait speed, the AUC curve for detecting an MCID of ≥0.05 m/s was similar to the primary analysis (S1 Table). The Youden Index identified a cut point of ≥3 steps/min, with a sensitivity of 0.81 and specificity of 0.88. For the 6MWT, walking cadence changes also demonstrated similar predictive accuracy for alternate MCID thresholds of ≥50 meters and ≥20 meters (S2 Table). The optimal cut point for detecting a ≥ 50-meter improvement was ≥ 4 steps/min, with a sensitivity of 0.81 and specificity of 0.80. For a ≥ 20-meter improvement, the Youden cut point was ≥ 3 steps/min, with a sensitivity of 0.75 and specificity of 0.81.

In the second sensitivity analysis, multivariable models incorporating age, body mass index, sex, and baseline walking performance demonstrated similar discrimination to the univariable models. For usual-pace gait speed, the AUC for detecting an MCID of ≥0.1 m/s was comparable to the primary analysis, and similar findings were observed for the 6MWT at an MCID of ≥30 m (S3 Table).

## Discussion

In this secondary analysis of an exercise intervention trial in older adults, we found that changes in walking cadence, measured during both usual pace gait speed and the 6MWT, were strong predictors of meaningful functional improvement. An increase in walking cadence of ≥3 steps/min during usual-pace gait speed was associated with improvements exceeding 0.1 m/s—the widely accepted MCID for gait speed—and similarly, a ≥ 3 steps/min cadence increase during the 6MWT accurately identified participants who surpassed the MCID (>30 m) for 6MWT distance. These findings were robust across a range of MCID thresholds in our sensitivity analysis and suggest cadence can be a practical and objective marker of exercise responsiveness in older adults.

Walking cadence is an intuitive and accessible parameter to track changes in physical function, as walking cadence is responsive to exercise interventions. Because increases in walking speed or distance require changes in either cadence (steps per minute) or stride length, cadence in this context should be viewed as a practical proxy for gait speed rather than a distinct construct. This relationship makes cadence a scalable method to approximate clinically meaningful changes in gait speed through remotely measured changes in step frequency. Tudor-Locke and colleagues recently demonstrated that increases in cadence among older adults were associated with higher energy expenditure, underscoring cadence as a modifiable and meaningful metric [7]. Although prior studies have characterized cadence in specific populations, few have evaluated how cadence changes in response to exercise interventions [20]. Among the limited studies that have examined walking cadence during a 6MWT, older adults with COPD exhibited lower cadence and shorter distances than healthy older adults, highlighting the burden of disease on walking performance [21]. However, that study did not evaluate changes in cadence or link those changes to intervention response. Our study fills this gap by demonstrating that improvements in cadence during the 6MWT can identify those who improved their walking endurance.

Our findings for usual pace gait speed reinforce prior literature demonstrating that cadence is responsive to exercise interventions. For example, Lord et al. showed in a 22-week aerobic and strength training intervention that older women improved cadence during usual pace walking [22]. Participants in the exercise group demonstrated a mean increase of 0.06 m/s with an associated mean increase in cadence of 2.6 steps/min. Notably, participants who demonstrated a reduced cadence at baseline were more likely to increase their walking speed. Similarly, Schwenk et al. found that a 12-week resistance training program in older adults with dementia increased cadence in older adults [23]. Our study extends these findings by linking changes in usual pace cadence to clinically meaningful improvements in gait speed, suggesting that cadence is a sensitive marker for capturing subtle but important functional gains.

These findings have important implications for research and clinical care. The usual pace gait speed and 6MWT are widely used endpoints to assess treatment response in older adults, but in-person functional assessments are often time- and resource-intensive. Smartphone and smartwatch-based platforms can repeatedly deliver standardized walk tests and capture changes in cadence during these assessments, enabling remote evaluation of functional change. Our study directly links changes in walking cadence to clinically meaningful improvements in usual pace gait speed and 6MWT performance and provides a framework for interpreting cadence within an MCID-based responder/non-responder paradigm. This approach advances prior work by moving cadence from a descriptive metric to a clinically interpretable digital endpoint that can be used in real-world settings. This has particular relevance for underserved and rural populations, as well as telehealth based care, where access to in-person testing is limited and clinicians may benefit from objective, remotely collected measures of function. In research settings, using cadence to approximate 6MWT or gait speed response could reduce the logistical burden of in-person follow-ups, improve retention, and lower study costs—especially in large-scale trials or epidemiologic cohorts. Notably, the American Heart Association has identified functional capacity, commonly assessed using the 6MWT, as a key endpoint for therapies in older adults with cardiovascular disease, further underscoring the relevance of scalable, cadence-based digital endpoints [24].

While our study has several strengths, including a well-characterized cohort and standardized assessments, we acknowledge several limitations. First, all functional assessments were conducted in a supervised laboratory environment, which may limit generalizability to unsupervised or home-based settings. Participants often perform better during supervised tests, and remote assessments may not yield the same predictive accuracy [25]. Future work is needed to evaluate whether cadence changes during at-home 6MWT or usual pace gait speed reflect true functional improvements. Second, the study population was predominantly White and relatively high functioning. Although participants were at risk for mobility decline, the mean gait speed was approximately 1.0 m/s and the average Short Physical Performance Battery (SPPB) score was approximately 10. As such, our approach will need to be validated in more frail populations with slower baseline walking speeds and in a more diverse cohort. Third, while our intervention led to measurable improvements in cadence, the generalizability of cadence changes across different types of interventions remains to be determined. Fourth, the identified threshold of a 3 steps/min increase in cadence to classify responders should be considered provisional and requires validation in independent samples, particularly in remote or unsupervised settings. Finally, the accuracy and consistency of wearable-derived cadence measurements in real-world environments remain important considerations. While this study used research-grade ActiGraph accelerometers, the performance of commercial wearable devices varies and is often influenced by proprietary algorithms. Continued validation of cadence measurement across devices and settings, along with greater transparency in analytic approaches, will be important to support the broader application of cadence as a digital endpoint.

In conclusion, we identified that increases in walking cadence during both the 6MWT and usual pace gait speed were associated with clinically meaningful improvements in walking function among older adults undergoing an exercise intervention. A provsional threshold of ≥3 steps/min increase in cadence may serve as an effective cut point for identifying responders, offering a promising, scalable approach to functional assessment. Further research is warranted to validate these findings in remote settings and across different interventions. If confirmed, cadence may enable the remote monitoring of physical function in older adults, supporting both clinical decision-making and research aimed at improving mobility and independence.

## Supporting information

S1 Table Univariable Logistic Regression results for MCID 0.05m/s usual pace gait speed. (DOCX)

S2 Table Univariable Logistic Regression results for 6MWT MCID ≥50 and ≥20 meters. (DOCX)

S3 Table Multivariable Logistic Regression results for usual pace gait speed (≥0.1m/s) and 6MWT (≥30 meters). (DOCX)

## References

[1] Harada ND, Chiu V, Stewart AL. Mobility-related function in older adults: assessment with a 6-minute walk test. Arch Phys Med Rehabil. 1999;80(7):837–41. doi: 10.1016/s0003-9993(99)90236-8 10414771

[2] Studenski S, Perera S, Patel K, Rosano C, Faulkner K, Inzitari M, et al. Gait speed and survival in older adults. JAMA. 2011;305(1):50–8. doi: 10.1001/jama.2010.1923 21205966 PMC3080184

[3] Arena R, Myers J, Williams MA, Gulati M, Kligfield P, Balady GJ, et al. Assessment of functional capacity in clinical and research settings: a scientific statement from the American Heart Association Committee on Exercise, Rehabilitation, and Prevention of the Council on Clinical Cardiology and the Council on Cardiovascular Nursing. Circulation. 2007;116(3):329–43. doi: 10.1161/CIRCULATIONAHA.106.184461 17576872

[4] Dibartolo MC, McCrone S. Recruitment of rural community-dwelling older adults: barriers, challenges, and strategies. Aging Ment Health. 2003;7(2):75–82. doi: 10.1080/1360786031000072295 12745386

[5] Hensen B, Mackworth-Young CRS, Simwinga M, Abdelmagid N, Banda J, Mavodza C, et al. Remote data collection for public health research in a COVID-19 era: ethical implications, challenges and opportunities. Health Policy Plan. 2021;36(3):360–8. doi: 10.1093/heapol/czaa158 33881138 PMC7928874

[6] Rubin DS, Straczkiewicz M, Yamamoto E, Madariaga MLL, Ferguson M, Brach JS, et al. A Smartphone Application to Measure Walking Cadence before Major Abdominal Surgery in Older Adults. Digit Biomark. 2025;9(1):113–23. doi: 10.1159/000545982 40636779 PMC12240576

[7] Tudor-Locke C, Mora-Gonzalez J, Ducharme SW, Aguiar EJ, Schuna JM Jr, Barreira TV, et al. Walking cadence (steps/min) and intensity in 61-85-year-old adults: the CADENCE-Adults study. Int J Behav Nutr Phys Act. 2021;18(1):129. doi: 10.1186/s12966-021-01199-4 34556146 PMC8461976

[8] Straczkiewicz M, Keating NL, Schonholz SM, Matulonis UA, Horowitz N, Campos S, et al. Smartphone-Based Measures as Indicators of Functional Status in Patients With Advanced Cancer. JAMA Netw Open. 2025;8(9):e2532488. doi: 10.1001/jamanetworkopen.2025.32488 40965888 PMC12447243

[9] Rubin DS, Hung A, Yamamoto E, Hedeker D, Conroy DE, Huisingh-Scheetz M, et al. Walking cadence as a measure of activity intensity and impact on functional capacity for prefrail and frail older adults. PLoS One. 2025;20(7):e0323759. doi: 10.1371/journal.pone.0323759 40668771 PMC12266393

[10] Bohannon RW, Crouch R. Minimal clinically important difference for change in 6-minute walk test distance of adults with pathology: a systematic review. J Eval Clin Pract. 2017;23(2):377–81. doi: 10.1111/jep.12629 27592691

[11] Brach JS, VanSwearingen JM, Gil A, Nadkarni NK, Kriska A, Cham R, et al. Program to improve mobility in aging (PRIMA) study: Methods and rationale of a task-oriented motor learning exercise program. Contemp Clin Trials. 2020;89:105912. doi: 10.1016/j.cct.2019.105912 31838258 PMC6945812

[12] Brach JS, Perera S, Shuman V, Gil AB, Kriska A, Nadkarni NK, et al. Effect of Timing and Coordination Training on Mobility and Physical Activity Among Community-Dwelling Older Adults: A Randomized Clinical Trial. JAMA Netw Open. 2022;5(5):e2212921. doi: 10.1001/jamanetworkopen.2022.12921 35604689 PMC9127558

[13] ATS Committee on Proficiency Standards for Clinical Pulmonary Function Laboratories. ATS statement: guidelines for the six-minute walk test. Am J Respir Crit Care Med. 2002;166(1):111–7. doi: 10.1164/ajrccm.166.1.at1102 12091180

[14] Guralnik JM, Ferrucci L, Simonsick EM, Salive ME, Wallace RB. Lower-extremity function in persons over the age of 70 years as a predictor of subsequent disability. N Engl J Med. 1995;332(9):556–61. doi: 10.1056/NEJM199503023320902 7838189 PMC9828188

[15] Berg KO, Wood-Dauphinee SL, Williams JI, Maki B. Measuring balance in the elderly: validation of an instrument. Can J Public Health. 1992;83 Suppl 2:S7–11. 1468055

[16] Karas M, Stra Czkiewicz M, Fadel W, Harezlak J, Crainiceanu CM, Urbanek JK. Adaptive empirical pattern transformation (ADEPT) with application to walking stride segmentation. Biostatistics. 2021;22(2):331–47. doi: 10.1093/biostatistics/kxz033 31545345 PMC8036002

[17] Karas M, Urbanek JK, Illiano VP, Bogaarts G, Crainiceanu CM, Dorn JF. Estimation of free-living walking cadence from wrist-worn sensor accelerometry data and its association with SF-36 quality of life scores. Physiol Meas. 2021;42(6). doi: 10.1088/1361-6579/ac067b 34049292

[18] Perera S, Mody SH, Woodman RC, Studenski SA. Meaningful change and responsiveness in common physical performance measures in older adults. J Am Geriatr Soc. 2006;54(5):743–9. doi: 10.1111/j.1532-5415.2006.00701.x 16696738

[19] Guralnik J, Bandeen-Roche K, Bhasin SAR, Eremenco S, Landi F, Muscedere J, et al. Clinically Meaningful Change for Physical Performance: Perspectives of the ICFSR Task Force. J Frailty Aging. 2020;9(1):9–13. doi: 10.14283/jfa.2019.33 32150208 PMC7286121

[20] Annegarn J, Spruit MA, Savelberg HHCM, Willems PJB, van de Bool C, Schols AMWJ, et al. Differences in walking pattern during 6-min walk test between patients with COPD and healthy subjects. PLoS One. 2012;7(5):e37329. doi: 10.1371/journal.pone.0037329 22624017 PMC3356256

[21] Liu W-Y, Spruit MA, Delbressine JM, Willems PJ, Yentes JM, Bruijn SM, et al. Alterations in stride-to-stride fluctuations in patients with chronic obstructive pulmonary disease during a self-paced treadmill 6-minute walk test. PLoS One. 2024;19(3):e0300592. doi: 10.1371/journal.pone.0300592 38489297 PMC10942081

[22] Lord SR, Lloyd DG, Nirui M, Raymond J, Williams P, Stewart RA. The effect of exercise on gait patterns in older women: a randomized controlled trial. J Gerontol A Biol Sci Med Sci. 1996;51(2):M64–70. doi: 10.1093/gerona/51a.2.m64 8612105

[23] Schwenk M, Zieschang T, Englert S, Grewal G, Najafi B, Hauer K. Improvements in gait characteristics after intensive resistance and functional training in people with dementia: a randomised controlled trial. BMC Geriatr. 2014;14:73. doi: 10.1186/1471-2318-14-73 24924703 PMC4062767

[24] Forman DE, Arena R, Boxer R, Dolansky MA, Eng JJ, Fleg JL, et al. Prioritizing Functional Capacity as a Principal End Point for Therapies Oriented to Older Adults With Cardiovascular Disease: A Scientific Statement for Healthcare Professionals From the American Heart Association. Circulation. 2017;135(16):e894–918. doi: 10.1161/CIR.0000000000000483 28336790 PMC7252210

[25] Urbanek JK, Roth DL, Karas M, Wanigatunga AA, Mitchell CM, Juraschek SP. Free-Living Gait Cadence Measured by Wearable Accelerometer: A Promising Alternative to Traditional Measures of Mobility for Assessing Fall Risk. The Journals of Gerontology Series A, Biological Sciences and Medical Sciences. 2023;78(5):802–10. 35029661 10.1093/gerona/glac013 PMC10172982

