# Supplementary material for: Change in walking cadence as a digital outcome measure of clinically meaningful improvement in gait speed and 6-minute walk test distance after a mobility intervention in older adults
Source: PLoS One. 2026 May 29;21(5):e0337414. doi: 10.1371/journal.pone.0337414 (PMC13221036; doi:10.1371/journal.pone.0337414)
Supplement: S1 Table — (DOCX) [file pone.0337414.s001.docx]

**S1:** Univariable Logistic Regression results for MCID 0.05m/s usual pace gait speed

| **S1: Univariable Logistic Regression** | | | |
| --- | --- | --- | --- |
| **Dependent variable: Usual Pace Walk Test (MCID 0.05m/s)** | | | |
| Predictor | Odds Ratio (per 1 step/min increase) | 95% Confidence Interval | P-value |
| Change in cadence during usual pace walk test | 1.71 | 1.47, 1.98 | <0.01 |
| Intercept | 0.34 | 0.20, 0.56 | <0.01 |
| AUC (95% CI) | 0.92 | 0.88, 0.96 | - |
