# Supplementary material for: Change in walking cadence as a digital outcome measure of clinically meaningful improvement in gait speed and 6-minute walk test distance after a mobility intervention in older adults
Source: PLoS One. 2026 May 29;21(5):e0337414. doi: 10.1371/journal.pone.0337414 (PMC13221036; doi:10.1371/journal.pone.0337414)
Supplement: S2 Table — (DOCX) [file pone.0337414.s002.docx]

**S2:** Univariable Logistic Regression results for 6MWT MCID >50 and >20 meters

| **S2: Univariable Logistic Regression** | | | |
| --- | --- | --- | --- |
| **Dependent variable: 6MWT (MCID >50 meters)** | | | |
| Predictor | Odds Ratio (per 1 step/min increase) | 95% Confidence Interval | P-value |
| Change in cadence during 6MWT | 1.23 | 1.14, 1.32 | <0.01 |
| Intercept | 0.24 | 0.15, 0.37 | <0.01 |
| AUC (95% CI) | 0.85 | 0.79, 0.91 | - |
|  |  |  |  |
| **Univariable Logistic Regression** | | | |
| **Dependent variable: 6MWT (MCID >20 meters)** | | | |
| Predictor | Odds Ratio (per 1 step/min increase) | 95% Confidence Interval | P-value |
| Change in cadence during 6MWT | 1.19 | 1.11, 1.27 | <0.01 |
| Intercept | 1.16 | 0.82, 1.65 | <0.01 |
| AUC (95 % CI) | 0.84 | 0.79, 0.89 | - |
