# Supplementary material for: Change in walking cadence as a digital outcome measure of clinically meaningful improvement in gait speed and 6-minute walk test distance after a mobility intervention in older adults
Source: PLoS One. 2026 May 29;21(5):e0337414. doi: 10.1371/journal.pone.0337414 (PMC13221036; doi:10.1371/journal.pone.0337414)
Supplement: S3 Table — (DOCX) [file pone.0337414.s003.docx]

**S3**. Multivariable Logistic Regression results for usual pace gait speed (>0.1m/s) and 6MWT (>30 meters)

| **S3: Multivariate Logistic Regression** | | | |
| --- | --- | --- | --- |
| **Dependent variable: Usual Pace Walk Test (MCID >0.1 m/s)** | | | |
| Predictor | Odds Ratio (per 1 step/min increase) | 95% (Confidence Interval) | P-value |
| Change in cadence during usual-pace walk test | 1.55 | 1.37, 1.76 | <0.01 |
| Age | 0.97 | 0.90, 1.04 | 0.34 |
| Body Mass Index | 1.01 | 0.93, 1.09 | 0.86 |
| Sex (Ref: Female) | 0.57 | 0.25, 1.28 | 0.17 |
| Baseline gait speed | 1.15 | 0.08, 16.72 | 0.92 |
| Intercept | 0.12 | 0.06, 0.22 | <0.01 |
| Model AUC | 0.91 | 0.86, 0.95 | - |
|  | | | |
| **Dependent variable: 6-minute walk test (MCID > 30meters)** | | | |
| Predictor | Odds Ratio (per 1 step/min increase) | 95% (Confidence Interval) | P-value |
| Change in cadence during 6-minute walk test | 1.18 | 1.11, 1.25 | <0.01 |
| Age | 0.90 | 0.85, 0.96 | 0.001 |
| Body Mass Index | 0.91 | 0.85, 0.97 | 0.004 |
| Sex (Ref: Female) | 0.69 | 0.35, 1.36 | 0.28 |
| Baseline 6MWT distance | 0.99 | 0.99, 1 | 0.001 |
| Intercept | 0.68 | 0.48, 0.98 | 0.05 |
| Model AUC | 0.80 | 0.74, 0.86 | - |
